# Peripheral arterial disease diagnosis and management in primary care: a qualitative study

**DOI:** 10.3399/bjgpopen19X101659

**Published:** 2019-08-21

**Authors:** Jan Lecouturier, Jason Scott, Nikki Rousseau, Gerard Stansby, Andrew Sims, John Allen

**Affiliations:** 1 Senior Research Associate, Institute of Health & Society, Newcastle University, Newcastle upon Tyne, UK; 2 Senior Lecturer, Faculty of Health & Life Sciences, Northumbria University, Newcastle upon Tyne, UK; 3 Senior Research Methodologist, Institute of Health & Society, University of Newcastle upon Tyne, Newcastle upon Tyne, UK; 4 Professor of Vascular Surgery, Northern Vascular Centre, Freeman Hospital, Newcastle upon Tyne Hospitals NHS Foundation Trust, Newcastle upon Tyne, UK; 5 Head of Department, Northern Medical Physics and Cliniical Engineering, Newcastle upon Tyne Hospitals NHS Foundation Trust, Newcastle upon Tyne, UK; 6 Honorary Senior Lecturer, Faculty of Medical Sciences, Newcastle University, Newcastle upon Tyne, UK; 7 Lead Clinical Scientist in Microvascular Diagnostics, Northern Medical Physics and Clinical Engineering, Newcastle upon Tyne Hospitals NHS Foundation Trust, Newcastle upon Tyne, UK

**Keywords:** Diagnosis, Qualitative research, General practice, Primary care, Peripheral arterial disease

## Abstract

**Background:**

Patients diagnosed with peripheral arterial disease (PAD) are at an increased risk of coronary heart disease, stroke, heart attack, and PAD progression. If diagnosed early, cardiovascular risk factors can be treated and the risk of other cardiovascular diseases can be reduced. There are clear guidelines on PAD diagnosis and management, but little is known about the issues faced in primary care with regards adherence to these, and about the impact of these issues on patients.

**Aim:**

To identify the issues for primary care health professionals (HPs) and patients in PAD diagnosis and management, and to explore the impact of these on HPs and PAD patients.

**Design & setting:**

Qualitative study conducted in a primary care setting in the North East of England. Data was collected between December 2014 and July 2017.

**Method:**

Semi-structured interviews and focus groups were conducted with PAD register patients (*n* = 17), practice nurses ([PNs], *n* = 17), district nurses (DNs], *n* = 20), tissue viability nurses (*n* = 21), and GPs (*n* = 21).

**Results:**

HPs’ attitudes to PAD, difficulty accessing tests, and patient delays impacted upon diagnosis. Some HPs had a reactive approach to PAD identification. Patients lacked understanding about PAD and some reported a delay consulting their GP after the onset of PAD symptoms. After diagnosis, few were attending for regular GP follow-up.

**Conclusion:**

Patient education about PAD symptoms and risks, and questioning about exercise tolerance, could address the problem of under-reporting. Annual reviews could provide an opportunity to probe for PAD symptoms and highlight those requiring further investigation. Improved information when PAD is diagnosed and, considering the propensity for patients to tolerate worsening symptoms, the introduction of annual follow-up (at minimum) is warranted.

## How this fits in

This article addresses the gap in the literature on the experience of primary care HPs and patient perspectives on the diagnosis and management of PAD. It gives weight to the international literature demonstrating the difficulties that patients and primary care HPs experience in relation to PAD diagnosis and management, and uncovers issues that could explain the delayed diagnosis and under-treating of PAD. It highlights the need for: improved information to PAD patients and the introduction of regular follow-up; a more proactive attitude to PAD identification by probing for symptoms during routine interactions; and greater adherence to PAD guidelines.

## Introduction

A diagnosis of PAD indicates an increased risk of coronary heart disease, stroke, and heart attack.^1^ Symptoms include intermittent claudication, which is calf pain on walking. One complication of PAD is critical limb ischaemia, where patients may progress to: severe pain at rest, usually felt in the foot; open wounds that are difficult to heal; and gangrene.^2^ As a large proportion of patients with PAD are asymptomatic or minimally symptomatic , the condition is often under-reported and under-treated.^3^ Symptomatic patients often delay consulting their doctor and present when the condition is at an advanced stage.^4^

For PAD diagnosis and assessment, clinical examination, detailed history, and ankle brachial pressure index (ABPI) testing with a handheld Doppler ultrasound probe are recommended.^5^ Though PAD is incurable, cardiovascular risk can be reduced with lifestyle changes (smoking cessation, weight control, and exercise) and medication. However, there is evidence of low compliance with PAD management guidelines such as lipid^6^ and blood pressure control.^7^ Supervised exercise is recommended for all patients,^5^ but very few UK clinical commissioning groups provide this service.^8^ Patients with mild PAD should be managed in primary care; if the condition worsens or does not resolve, or if there are problems with diagnosis, they should be referred to secondary care.^5^

There is little published UK-based research on patients’ and primary care HPs’ experiences of PAD diagnosis and management. This article reports the findings from one work package of a large programme of research around technology to diagnose PAD. This was a qualitative study of patients, community nurses, and GPs to identify the issues they face in PAD diagnosis and management in primary care, and the impact of these issues.

## Method

Qualitative methods (interviews and focus groups) were used to identify the issues from the perspectives of the study participants. To avoid imposing known issues around the diagnosis and management of PAD from the literature and from the experience of the clinical team, broad topic guides were used that covered patients' history of PAD, experience of diagnosing or being diagnosed with PAD, and how PAD is managed (topic guides are available from the authors on request). Broad topic guides enabled the participants to raise issues themselves rather than issues being suggested by the researchers.

This research was conducted in a primary care setting in the North East of England. Patients from the PAD register (the National Institute for Health and Care Excellence (NICE) Quality and Outcomes Framework (QOF) Indicator states that general practices should establish and maintain a register of patients with PAD) and PNs from the practices participating in the diagnostic study (to compare ABPI, innovative multi-site photoplethysmography technology, and a duplex ultrasound arterial scan) were approached to participate in a face-to-face or telephone interview. For focus groups, a purposive sample was drawn of: small and larger general practices (including GPs, PNs, DNs) in areas where PAD would be more prevalent; tissue viability nurses; and separate DN groups. Supplementary telephone interviews were conducted with PNs and GPs from other regional general practices to explore in greater depth the issues that were raised in the focus groups and interviews.

Participants were provided with a study information sheet and signed a consent form. Interviews and focus groups were digitally recorded with participant consent. Recordings were transcribed, anonymised, and managed using NVivo (version 11). All were analysed using a thematic approach (Table 1).^9^ JS generated the initial themes which were iteratively developed in data sessions with JL and NR.

**Table 1.** Steps in data analysis

**Step 1: Familiarisation with data** — read, re-read, and listen to recordings of interviews/focus groups
**Step 2: Generate initial codes** — systematically record features of the data that are interesting across the data
**Step 3: Identify themes** — sort code extracts into overarching themes. Develop subthemes where appropriate
**Step 4: Review of themes** — themes are combined, refined, redefined, or separated. From this map, a framework is devised
**Step 5: Defining and naming themes** — another stage of refinement of the themes and subthemes; concise working definitions of each theme are added

## Results

**Figure 1.**
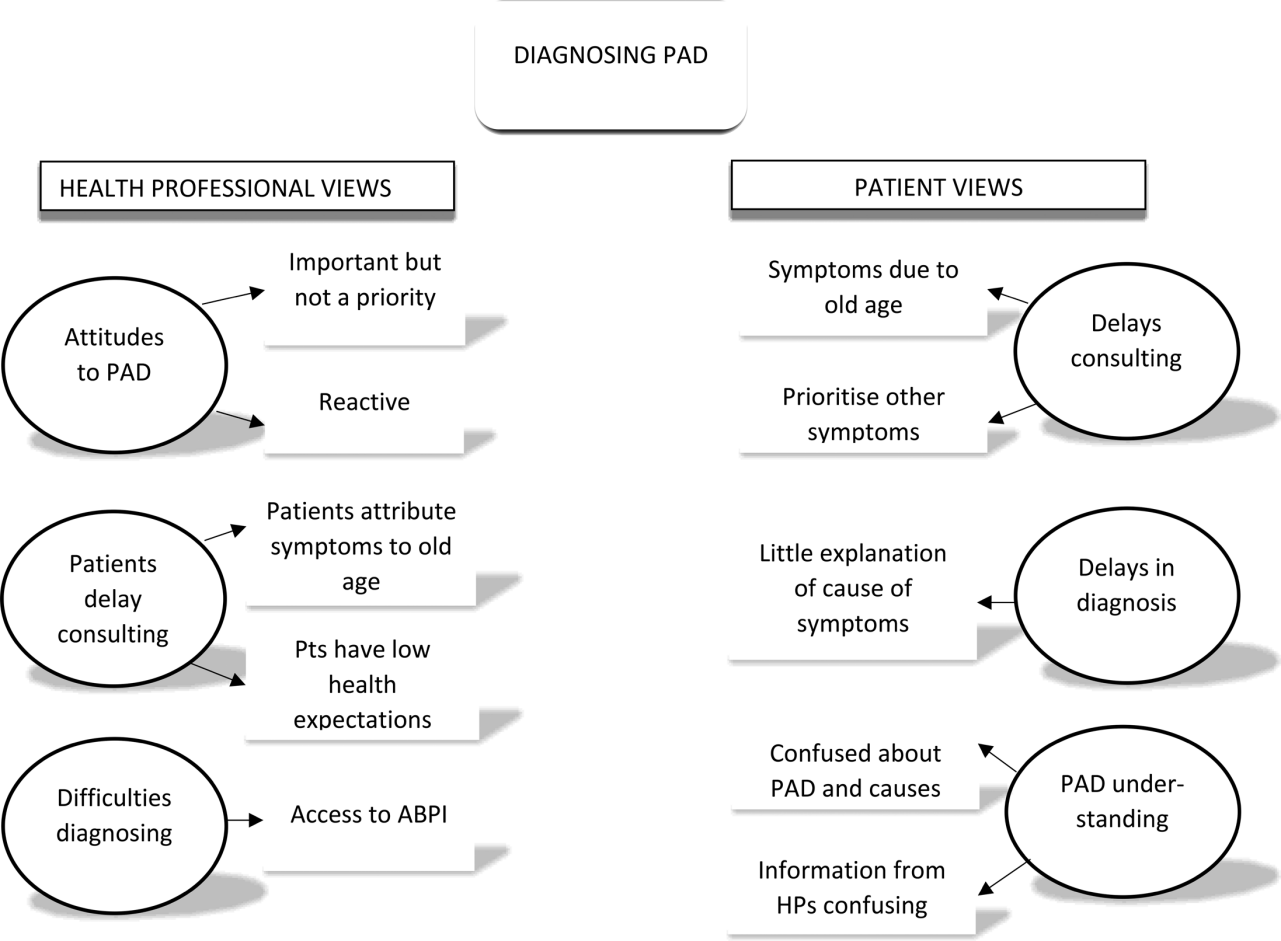
Themes: diagnosing PAD ABPI = ankle brachial pressure index. HP = health professionals. PAD = peripheral arterial disease. Pts = patients.

**Figure 2.**
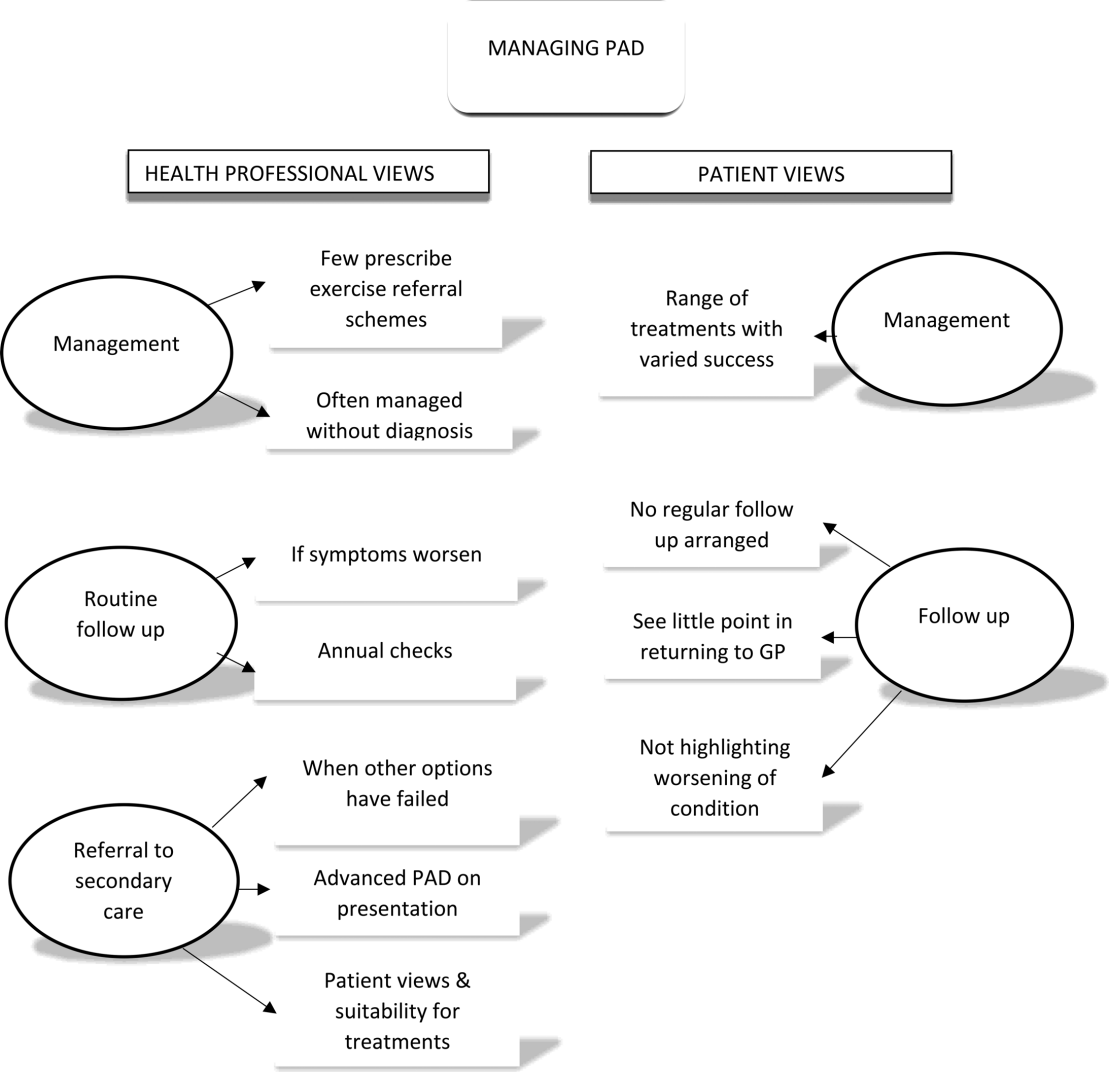
Themes: managing peripheral arterial disease (PAD)

Data collection began in December 2014 and ended in July 2017. A total of 96 nurses, GPs, and patients with PAD participated: 17 PNs; 20 DNs; 21 tissue viability nurses; 21 GPs; and 17 patients with PAD. The key themes elicited from the data are outlined in Figures 1 and 2. Quotations are presented with participant identifier codes, including ‘FG’ for focus group participants and ‘Int’ for interview participants, and abbreviations for participant type (for example, ‘DN’ for district nurse and ‘Pt’ for patient).

### Diagnosing peripheral arterial disease

#### HP perspectives

##### Attitudes to PAD

HPs recognised the importance of diagnosing PAD and understood that early identification can facilitate secondary prevention of cardiovascular disease. Only a few HPs mentioned that identification provided an opportunity to manage and prevent the progression of the often debilitating symptoms of PAD:

*‘* *If you start battling it with antiplatelet and lowering cholesterol and making sure the blood pressure’s on the button, you’ll do* [patients] *some huge favours, not just in terms of walking distance but in terms of not having a heart attack or a stroke. And there is another group of patients I’ve only really become more aware of recently* […] *where their skin is going to break down, they’re going to get ulcers and you know* […] *to get a chronic leg ulcer is disastrous.* *’* (Int/GP18)

Although PAD was considered to have ‘*gained prominence in recent* *years*’ (FG/GP4) no one mentioned actively looking for it, instead describing a reactive rather than proactive approach:

*‘* [PAD] *probably is a relevant thing to be looking for and I’m sure it is a marker of vascular disease and worthy of treatment* […] *we’re not actively searching people out, so we are only really responding to* […] *the symptoms that people present rather than actively looking for it*.’ (FG/GP1)

PAD diagnosis was not considered a priority for the majority of HPs. PAD testing is not currently part of the QOF, and for some GPs other conditions took precedence: ‘S*tuff that doesn’t have a QOF domain gets sort of sidelined a bit’* (Int/GP3). Also, in comparison to other conditions, there is no immediate effect of PAD interventions: ‘*you don’t notice how much good work you do in your practice by stopping somebody having a stroke because you don’t know you’ve stopped them having a stroke or a heart attack*’ (Int/GP18). It was suggested that HPs can have a fatalistic attitude to PAD and, as a consequence, its identification is not a priority.

For those with a more proactive approach, the focus was on patients at a higher risk of PAD. One GP described an unstructured approach of questioning to identify intermittent claudication, but acknowledged the difficulties of doing so in a 10 minute consultation:

*‘* *It will be those patients with multiple other pathologies who are carrying PAD and because that’s not at the front of everybody’s mind, it can easily get ignored and forgotten.’* (Int/GP18)

##### Delays in patients consulting the GP

Sometimes PAD symptoms were identified when patients consulted an HP for another condition, or patients became aware when they were required to walk more because of changes in their personal lives. It was felt patients lacked of awareness of PAD symptoms and risks: ‘*I don’t think they necessarily associate it with heart disease.’* (Int/HP7). District and practice nurses commented that some patients, in the face of obvious changes and symptoms, delay consulting their doctor:

*‘Often you see patients, the GP has had no input into them at all* […] *they don’t understand what’s happening. No hairs on their legs and the ankles starting to waste a bit and they get pain when they walk. But they don’t see their GP until way down the line.’* (FG/DN1)

The reasons for delaying were thought to be due to a tendency for patients to attribute symptoms to getting older, and also to low health expectations in areas of greater deprivation:

*‘For the sort of folk that I’m looking after, maybe … you know, the majority of them will have left school at 16, not much further education and the generation that they* […] *look to* […] *their parents with PAD* […] *Their expectations of health are very low.’* (Int/GP18)

##### Difficulty diagnosing

When patients do present, HPs reported using a variable combination of symptoms, history, and ABPI to diagnose PAD. There was variation in the value individual GPs placed on ABPI in the PAD diagnostic pathway. Some relied on clinical judgment based on reported symptoms and a physical assessment, and expected ABPI would be conducted (to a higher standard) when the patient was eventually referred to secondary care. ABPI would not be conducted when patients with suspected PAD were asymptomatic. Based on the presentation of symptoms, one point at which some HPs would consider it futile to conduct ABPI was the very same point others thought it necessary:

*‘These people tend to come in* […] *and if they’ve got an ice cold foot and you think they’ve got an ischaemic leg, you’re not going to worry about ABPIs at that stage.’* (FG/GP2)

*‘So any of the spectrum of symptoms* […] *up to the very extremes; pain, perishingly cold, ulcers, pale* […] *I think most of us would look at them in the same way, once we had ruled out an acute ischaemic episode, we’d probably consider getting ABPIs at that point.’* (FG/GP3)

ABPI was the most commonly mentioned problem around diagnosing PAD. One GP thought the main challenge was lack of access to ABPI and the waiting time where it was available. Three routes to an ABPI test for patients with suspected PAD were described: PN; referral to the district nursing service; and referral to the secondary care vascular team. In some areas, DNs were contracted to conduct ABPI only in leg ulcer patients; DNs said PNs should conduct ABPI for suspected PAD. However, the number of general practices where the PNs were trained in ABPI was low, possibly because the training was said to involve a 2-day course. The QOF was again mentioned, and that training and the time taken to conduct ABPI takes PNs away from chronic disease management:

*‘From the practice manager's point of view, why would they send someone to do that training? So that's taking them out of clinic, so all that is missed QOF, isn't it, to then go and train in something that you don't get any QOF, which is also still quite time-consuming to book people in for*.’ (FG/DN2)

Where there was an agreement with the NHS trust for DNs to conduct ABPI, there was often a long wait because of other demands on DNs which resulted in some GPs not referring:

*‘I hardly ever requested them after that, as I thought “Okay, this isn’t something that practically we can get done very easily.”’* (FG/GP1)

In certain cases, no access to ABPI resulted in GPs referring patients to the secondary care vascular team. One GP said if the symptoms warranted further investigation, and in the absence of an easy and timely route to ABPI, they may use a Doppler ultrasound probe to check for a pulse, ‘*then if it looks suspicious that they’ve got* [PAD] *they basically get referred to secondary care who do the duplex artery scanning’* (FG/GP1).

##### What HPs can do

As there is a propensity for patients to suffer in silence, some HPs believed it to be their responsibility to probe for PAD symptoms. One option could be to target those at greater risk of PAD, for example patients with diabetes and cardiovascular disease. Although time is an issue, one GP commented: ‘*It’s no good saying, “20 minute appointments would be so much better,” of course they would but … you’ve got to work with what you got*.’ (Int/GP18). Another opportunity mentioned was the annual review — if offered — when PNs could ask about PAD symptoms:

*‘* *What I’ve learnt is, by looking at guidelines, I need to listen to my patients more and actually ask them the right questions. I’m not asking them the right questions to draw them out about their conditions. I’m not asking specifically about their leg pain, how far they could walk without getting pain.* *’* (Int/PN12)

#### Patient perspectives

##### Delays in patients consulting GP and delays in diagnosis

Most patients had experienced symptoms indicative of PAD for a number of years. Some had ignored these or attributed them to something else, for example ‘aching legs’. This led to one patient requiring invasive treatment for their PAD:

*‘When I was walking I ended up with pain in my calf that I disregarded for a time, then I gradually got worse and I ended up having a stent put in.’* (Int/Pt3)

Patients with comorbid conditions reported prioritising what they discussed with their GP, which suggests they considered PAD symptoms to be of lower importance than those related to other conditions:

*‘I think I mentioned it to* [GP] *but I never went into it. I think it’s just with me being used to having it that I seem to suffer in silence about it.’* (Int/Pt10)

When patients had consulted their GP — some at the point when they first experienced symptoms and others later — a number of patients were left without any real explanation of what was the cause:

*‘I think it goes back 11 years now. I’d just retired and I’d joined a walking group and was beginning to get pain in my left hip. I saw the GP about this and he didn’t actually say at the time what he thought it was. He just advised me to keep going and stop and have a rest when I was walking and try to walk through it.’* (Int/Pt2*)*

Undergoing ABPI did not always mean that they received a diagnosis: one patient reported receiving no feedback after the test. For the following two years the leg pain persisted, and after a second test they were referred to a cardiovascular specialist in secondary care and received a diagnosis. Another patient, despite having undergone a stent procedure, remained unclear what was causing their leg pain. When asked what their GP had told them they said:

*‘Well* [laughter] *apparently I’m a mystery,* […] *the thing is it’s not constant, I can’t say that every time I go out it’s the same symptoms* […] *If it was constant, if it was the same thing constantly, he could understand it apparently.’* (Int/Pt15)

##### Understanding of PAD

A number of patients struggled to understand what PAD is, its risk, or its cause. Awareness of PAD was considered in light of other more commonly encountered health problems:

*‘I’ll be straight to the point, if a doctor said, “Well, you’ve also got arthritis.” Then you would understand, but I haven’t got a clue, but you see I talk to friends and they’ll say, “You’ve got arthritis, that’s all it is.” I say, “Well, my ruddy feet are icy cold, I’m bad at walking, and I have pain on my toes.” So, I don’t know the real answer.’* (Int/Pt26)

Even when HPs do provide information, it was clear that some patients had problems making sense of it:

*‘When people like myself go to see one of these consultants, obviously you listen to what they say, and when you come away, you’ve got to pick the bones out of it.’* (Int/Pt12)

### Management of PAD

#### HP perspectives

PAD patients received lifestyle advice: healthy eating, sensible alcohol intake, as well as *‘Stop smoking,* *k* *eep walking!* *”* (FG/GP1). Only a few HPs mentioned exercise referral schemes. It was said some patients expect PAD can be *‘* *fixed* *’* (Int/GP18) and are disappointed when surgery is not offered (Int/GP3). When lifestyle changes have failed and the symptoms are worsening, PAD patients are assessed to determine the most appropriate step. This may be medication, but one HP expressed concern that, at this point, the diagnosis of PAD is not based on full information:

*‘I will probably put them on aspirin or clopidogrel but I will have done all of that without properly assessing their vascular tree. I’m loading a huge amount onto a typical history and not feeling the pulse properly and maybe it’s a bit arrogant but I probably am diagnosing a lot of them on “story and not being able to feel a pulse” alone.’* (Int/GP18)

#### Follow-up

Patients with suspected PAD have the ‘safety net’ of returning if symptoms worsen (Int/GP3). In some patients a PAD diagnosis may highlight other problems that require intervention and this will determine how often they are seen. Most practices offered an annual check-up (not always with a PAD focus), and one described their nurse-led vascular clinic, where PAD patients have checks for diabetes, and renal function and cholesterol are measured. Patients are asked to come back sooner if they are having problems. Two others described a similar process of annual monitoring of PAD patients: neither conducted ABPI at these reviews, one because it is not *‘* *part of the review* *’* (Int/PN8) and another as they do not have a Doppler ultrasound probe. Another said they were in the process of refining their reviews to ensure that they provide *‘* *specific advice around their condition* *’* (Int/PN12) to PAD patients.

#### Referral to secondary care

There was a general consensus that patients are referred to secondary care when their PAD symptoms are severe and the treatments available in primary care are no longer effective. When ABPI is available, a *‘* *borderline* *’* result would prompt referral (Int/GP3). One practice refers a number of patients to secondary care because they present with more advanced PAD:

*‘What we don’t tend to get is people who are at a very mild stage where it’s a question of establishing diagnosis, they tend to come a bit later than that*.’ (FG/GP1)

Other factors are considered before referring to secondary care for potentially more invasive treatment. These include patients’ views on the potential procedures, or where the patient is older, not living independently, and may be a poor candidate for surgery:

‘*It’s ultimately, what, what’s it going to achieve, you know? If they’re very elderly and they’re not symptomatic with it a lot of the time the GPs will decide to just monitor and just kind of do annual reviews as opposed to, quote, “Send them to vascular.” You know, it just depends on the bigger picture*.’ (FG/DN1)

### Patient perspectives

#### Management in primary care

Two patients reported being told to exercise but no one mentioned having supervised exercise: one would have liked to know about other treatment options (Int/Pt13). Five patients had been prescribed antiplatelet and/or statin medication. One person disliked taking statins and often forgot. Most said medication had made little difference, though one person said the symptoms had not worsened. Only one patient, on a combination of antiplatelet and statins, thought medication had helped:

*‘Before I had the medication, I couldn’t walk more than, sort of, 100 yards. It was quite frightening, really. I’ve never had a problem since, I get the odd twinge but I can walk for miles still, now. I take the statins in the evening, the clopidogrel in the morning and I’ve never had any bother since. That was nearly 16 months ago now.’* (Int/Pt11)

Five had undergone a stent procedure. Unfortunately for three patients, this had failed to alleviate the symptoms in the longer term, though it is unknown whether they were made aware initially that this could be an outcome. Another (Int/Pt12) was referred for a stent but the consultant felt the procedure was not suitable and suggested medication, which the patient reported did not work.

#### Engagement with GP and experience of follow-up in primary care

Only one patient mentioned the opportunity for regular review — a 6-month medication review — at their general practice (Int/Pt11). For most, there appeared to be no regular follow-up; one patient had not seen their GP about their PAD for a year (Int/Pt13). Another patient relied upon other HPs to determine if their PAD was worsening; one considered it their own responsibility:

*‘I think as a patient, you should be the first to notice if anything is changing and then see your GP.’* (Int/Pt1)

The notion of ‘suffering in silence’ occurred again. One patient had not been forthright about the impact of the PAD symptoms nor asked if something could be done, despite now needing a walking aid:

*‘I don’t mention it to* [GP] [*…*] *She has mentioned it but it’s been not like, like a cause, I don’t know how to put it, but it’s just been dropped and forgot about, you know?’* (Int/Pt10)

A patient who was told by a consultant they did not require a procedure said they would return to see their GP if the symptoms *‘* *got so bad that I couldn’t walk* *’* (Int/Pt24). However, others (Int/Pt12 and Int/Pt22) had made a conscious decision not to return to their GP, especially after consultation with the secondary care vascular team. If a specialist could do little to help, going back to their GP was thought to be pointless. This meant they were not being followed up by either their GP or hospital specialist:

*‘I certainly haven’t discussed if there’s anything further that they can do for me. So that’s about the state of affairs at the moment.’* (Int/Pt12)

## Discussion

These findings indicate that a variety of factors impact on the diagnosis of PAD, including a range of HPs’ attitudes to PAD, difficulties accessing ABPI tests, and patient delays in consulting. General practices in this study had different referral pathways for ABPI; where access to ABPI was problematic, PAD diagnosis was often based on patients’ reported symptoms rather than objective clinical tests. HPs were clear about the management pathway but acknowledged the complexities with a patient group that often have comorbidities. Few were prescribing exercise programmes despite evidence of their benefits for intermittent claudication.^5,10,11^ No one mentioned the guidelines for PAD,^5^ which were published just prior to the wider study commencing.

The main issue for patients was obtaining a diagnosis and (once diagnosed) follow-up, which appeared to vary. Some patients were left in the dark about disease progression or rarely discussed it with their GP, believing little could be done. There was a lack of understanding about the condition; some patients said they had gained more information about PAD through their participation in the wider diagnostic study^12,13^ of which this qualitative research was one work package. Patients delayed consulting their GP and downplayed the importance of symptoms or attributed them to old age, which concurs with HP data. Although HPs reported offering annual review, only one patient in the present sample reported that they had attended for review.

Some health professionals believed patient education about PAD symptoms and risks, and screening could address the problem of under-reporting. Screening could be problematic if ABPI is part of the process but probing about exercise tolerance could identify those who warrant further investigation. Patient annual reviews could provide an opportunity to probe for PAD, but PAD requires a higher profile, for instance through a QOF indicator.

### Strengths and limitations

A limitation of this study was that the views of the GPs from the practices of the patient participants were not obtained; these may have shed some further light on their individual experiences of PAD diagnosis and management. However, this did enable the exploration of the diagnosis and management of PAD across a greater number of general practices, albeit from different perspectives.

### Comparison with existing literature

There is a substantial body of literature about the use of ABPI for PAD diagnosis. It requires training, to ensure proficiency,^12,14^ and when used by a skilled operator, ABPI has good sensitivity and specificity.^15^ Studies conducted in the Netherlands, however, have raised issues about the reliability of ABPI conducted in primary care.^16,17^ The majority of responders to a UK GP survey found ABPI useful for the diagnosis of PAD, but a large proportion reported staff availability, training, and time constraints as limitations.^18^ The literature suggests difficulties accessing ABPI is not unique to the current UK study; in Australia, Haig *et al* ^19^ reported that better education of GPs and improved access to ABPI or a *‘* *more time efficient cost* *’* would facilitate the earlier detection of PAD; in the US staff availability was a limitation for ABPI.^20^

Other studies have identified that patients with PAD symptoms delay consulting their GP.^21,22^ Davies *et al* reported one third of patients with debilitating symptoms of intermittent claudication had not consulted their GP,^21^ and suggest a need to raise public awareness of PAD. A low level of patient knowledge about PAD and its risks has been reported in the UK^21–23^ and the Netherlands.^24^ The present findings add weight to these studies, including describing additional challenges in the diagnosis and management pathways. A qualitative study involving patients with intermittent claudication reported that participants felt neglected by HPs, had no planned follow-up care, had little specific information, and were left with the impression that their condition did not warrant the same attention as others.^22^

A UK study exploring the utility of PAD screening (using ABPI and two risk assessment scales) in general practice concluded that routine screening identified too few cases.^21^ However, they proposed that health professionals could improve the identification of PAD by including questions about claudication in consultations.

### Implications for research and practice

There is a need for: better information to patients; general awareness-raising of PAD and associated risks; more accessible PAD diagnostics; and greater adherence to NICE guidelines, particularly around prescribing exercise programmes. Considering patients’ reticence about consulting their GP, HPs should routinely probe for PAD in their interactions with patients at risk to identify those requiring further investigation. Those diagnosed should have regular follow-up. Up to this point, there have been no research studies exploring the impact of enhanced information, raising awareness of PAD, or specific probing for PAD.
